# Competition for students in the digital era: scientific disciplines as drivers of study program innovation in Swiss higher education

**DOI:** 10.1007/s10734-025-01548-3

**Published:** 2025-10-17

**Authors:** Philippe Saner, Katja Rost

**Affiliations:** 1 Center for Higher Education and Science Studies, University of Zurich, Zürich, Switzerland; 2 Department of Sociology and Center for Higher Education and Science Studies, University of Zurich, Zürich, Switzerland

**Keywords:** Competition, Higher education, Disciplines, Digital transformation, Study programs

## Abstract

Competition between universities has become more intense and multilevel in recent years. Whereas previous studies have mainly looked at multilevel competition in research, this paper investigates competition among universities in academic teaching related to digital transformation. We use a database of study programs on digitalization offered by Swiss universities between 2017 and 2023 to examine the levels at which competition for students occurs. Results show that most competition is found within scientific disciplines, with weaker forms of competition observed at the university, canton, and university type levels. The number of digitalization-related study programs offered in a discipline at a specific university increases significantly if the number of such programs increases in that discipline at other universities. The findings suggest that disciplines are significant drivers of new degree programs in digitalization because program innovation happens at the department and faculty levels rather than the university level. Although all types of universities are positioning themselves for digital transformation in teaching, universities of applied sciences are proving particularly entrepreneurial in creating new offerings. The study highlights that competition for students is intensifying due to digitalization, changing student demands, and demographic trends, which may lead to a new market segmentation in which universities increasingly specialize in disciplines in which they excel.

## Introduction

Science has long been a competitive activity (Bourdieu, 1975; Hagstrom, 1974; Merton, 1973). Nevertheless, competition between universities has intensified and become multilevel in recent decades (Bloch, 2021; Brunsson & Wedlin, 2021; Kosmützky & Krücken, 2024; Krücken, 2021; Musselin, 2018). Competition no longer occurs only between individual researchers and between nation-states but between global, national, and regional entities (Hasse & Krücken, 2013; Hüther & Krücken, 2016). Research has focused on the intensification and transformation of competition in research and among research universities (Bloch et al., 2024; Krücken, 2017; Musselin, 2018). Competition at disciplinary level has largely been neglected in the literature on multilevel competition because most research investigates institutional behavior at the university level (Baker, 2025; Bloch, 2021; Bloch et al., 2024). Moreover, university curricula have received less attention, partly because academic writers typically concentrate on indicators of research excellence (Münch, 2014; Yudkevich et al., 2023). However, in many European higher education (HE) systems, performance-based funding (PBF) allocates a large proportion of universities’ income, and thus of disciplines within them, not by research excellence but by student numbers (Ambrosy, 2014; Dyrstad et al., 2024; Jongbloed & Vossensteyn, 2016; Jongbloed, 2010; Nygård, 2024). The most relevant actors in this context are not universities but scientific disciplines: communities of scholars that share some collective agency (Meier, 2023) to compete with other disciplines for students and other resources. In many HE systems, disciplines are organized into departments (Jacobs, 2014). Departments that innovate in their study program offerings and invest in the quality of teaching can over time attract more students and thus more resources. As a result, they can be expected to be better positioned in university decision-making processes and to participate in establishing the rules of the field (Kosmützky & Meier, 2025; Meier, 2023).

Digital transformation presents an interesting opportunity to investigate such dynamics in HE. New fields of study are emerging, and existing ones are being transformed, a situation that in many ways changes the relations between and within disciplines (Saner et al., 2023). Digitalization also rapidly affects student and labor market demands (Börner et al., 2018; Frey & Osborne, 2017). This increases demand for certain degree programs and disciplinary knowledge while others lose importance. Through PBF allocations, this divergent student demand is translated into increasing or decreasing state funding for certain disciplines. This divergence has been exacerbated by the arrival of low-birth cohorts in many OECD countries (OECD, 2008; Vincent-Lancrin, 2020) and stagnating public investment due to rising budget deficits in many European states (Jongbloed & Vossensteyn, 2001; Tandberg, 2010). Disciplines that can react quickly to changing student demands in the digital era will also be better able to assert their positions in the face of increasing competition for students.

This paper combines these insights and focuses on competition in teaching on study programs on digitalization. Although this process can be observed in various HE systems, this paper centers on Switzerland. We ask two questions: Is there competition between Swiss universities in academic teaching in the context of digital transformation? And if so, at what level does this competition take place? We use a dataset of study programs on digitalization offered by Swiss universities between 2017 and 2023 to investigate empirically whether and if so at which levels competition for students occurs. We examine the relationship between various independent variables and the number of digitalization study programs at Swiss universities. Our analysis shows that competition for students occurs within disciplines and to a lesser extent within universities. We discuss the consequences of this competition for the emerging categories in HE.

## Multilevel competition in higher education: disciplinary agency, performance-based funding, and digitalization

### The role of scientific disciplines

The role of scientific disciplines has rarely been discussed in the literature on multilevel competition in HE (Bloch, 2021; Brunsson & Wedlin, 2021; Kosmützky & Krücken, 2024; Krücken, 2021; Musselin, 2018). In the sociology of science, disciplines are conceptualized as scholarly communities or cultures that are organized around specific concepts, theories, and methods (Becher & Trowler, 2001). Against this backdrop, Frank Meier (2023) has recently advanced the “collective agency” of disciplines. Individual and organizational actors “act and speak in the name of their discipline”, for example in university decision-making processes, and therefore “act as representatives of their disciplines” (Meier, 2023, p. 279). Three “disciplinary reflexive interests” contribute to disciplines’ “collective agency”: securing the means of production and reproduction, ensuring disciplinary representation in decision-making processes, and seeking favorable rules of the game (2023, p. 281). The reproduction of knowledge through the creation and expansion of study programs is crucial both materially in the number of students and epistemically in the transmission of concepts, theories, and methods from one generation of students to the next. In the discipline-based departmental system at universities (Jacobs, 2014), a stable or growing interest among students in a particular discipline is one indication that this discipline is well positioned within a university. Such interest also ensures that the field receives consistent funding over time.

The capacity to engage in pertinent decision-making processes and participate in determining the rules of the game serves to foster a favorable environment for a discipline, or at the least, to prevent it from being placed at a disadvantage relative to other disciplines. Disciplines within a university constantly observe and react to developments in their competitors’ supply (Arora-Jonsson et al., 2020, 2021), such as the introduction of new degree programs, the creation of new professorships, and the establishment of research centers. We define competitors as the same disciplines in departments at other universities. The underlying assumption is that in HE systems with PBF allocation (Baker, 2025; Dyrstad et al., 2024; Jongbloed & Vossensteyn, 2001; Nygård, 2024), only those disciplines that can successfully attract enough students and thus financial resources will survive in the long run at a university.

#### Performance-based funding of higher education: Striving for student numbers

In many European countries, the state or federal states finance universities almost entirely (Jongbloed & Vossensteyn, 2016; Jongbloed, 2010). In recent decades, the introduction of new public management and the increasing availability of metrics and rankings have resulted in a shift toward PBF allocations at the nation-state level (Baker, 2025; Dyrstad et al., 2024; Jongbloed & Vossensteyn, 2001; Nygård, 2024). The basic aim is to increase universities’ efficiency and performance through performance linkage, transparent target achievement, and competition (Weibel et al., 2010). Such performance agreements vary considerably depending on the nation-state and type of university (de Boer et al., 2015). They are also multilevel, with universities usually receiving a global budget depending on performance, which is then divided by the universities’ decision-making bodies, such as university councils, among the individual departments, again according to performance criteria (Baker, 2025).

The assessment basis for the global budget that public universities receive directly from the state is decided largely by student numbers and degrees and less by the criteria considered important for research excellence, such as publications, citations, and third-party funding (Münch, 2014; Yudkevich et al., 2023). Even in research-oriented PBF systems (Baker, 2025), the financial contributions made for academic excellence only account for around 20% of universities’ global budgets. The rest is determined by student numbers (Hofmann & Janger, 2023). Student numbers are thus key for public universities. Importantly, calculating universities’ global budgets from student numbers is widespread internationally, particularly in Western Europe (Ambrosy, 2014; Dyrstad et al., 2024; Jongbloed & Vossensteyn, 2016; Jongbloed, 2010; Nygård, 2024).

Consequently, the outputs of competition desired by academics as participants differ fundamentally from those of politicians as organizers of competition (Arora-Jonsson et al., 2020, 2021). University management falls between two stools: as scientists, they are committed to academic performance. However, they depend on state funding and must therefore pay attention to student numbers. For a long time, this conflict of aims remained inconspicuous. First, performance agreements were new and had to be tested. Second, until the 1990 s, most countries’ state deficits were not too high, and sufficient funding was available. Third, and particularly important to our argument, student numbers at the universities and in the disciplines increased due to the rising numbers of high school graduates entering universities (Schofer & Meyer, 2005), and these were distributed more or less evenly across universities and disciplines. However, the combination of smaller university student populations (OECD, 2008; Vincent-Lancrin, 2020) and digitalization has altered the situation, and student numbers are becoming increasingly important in HE competition.

#### The disruptive shift through digitalization in higher education

Digitalization was long considered nothing more than a buzzword (Saner et al., 2023). In a Schumpeterian radical market disruption (Schumpeter, 2006), this has changed dramatically within the previous two decades. As argued by Christensen (2015), this underestimation of disruptive technologies is regularly observed in markets: The dominant market players initially underestimate the potential of new technologies, in particular because customer demand is still low, and are then overtaken by rapid technological progress because they have invested too little in these technologies. For universities, this change has massively fueled and intensified multilevel competition in both research and teaching performance. First, digital technologies have accelerated global flows and networks of ideas, knowledge, and money (Marginson, 2006). This effect was exacerbated by university closures during the COVID-19 pandemic (Williamson et al. 2021). Second, digital technologies have increased the visibility and pervasiveness of rankings. Third, the emergence of vast amounts of scientific data, immense computing capacities, and algorithmic procedures have led to new scientific fields such as digital humanities, data science, and computational social sciences and the transformation of existing ones such as digital law, digital marketing, and digital religion (Saner et al., 2023). Fourth, buzzwords such as artificial intelligence, big data, and cybersecurity have rapidly changed HE graduates’ expectations of (Börner et al., 2018). Finally, digitalization has enabled new actors to enter HE, such as for-profit universities, big tech companies, knowledge providers, and venture capital firms (Williamson, 2021).

Worldwide, universities respond to these challenges by increasing their activities in digitalization (Saner et al., 2023; Selwyn, 2014). They create new competence centers and chairs dedicated to these topics. Their students begin learning digital skills and pursuing new degrees in digital fields. They adopt digital strategies, establish digitalization offices, and appoint vice presidents for digitalization (Tratschin et al., 2023). They invest heavily in digital infrastructures and platforms for learning, teaching, research, and university management (Williamson, 2021). They enter new alliances for technological innovation with private companies, other research institutions, and state actors. This multitude of activities and strategies demonstrates the challenges of intensified competition between universities due to digitalization: What will prove sustainable in the future and what will fail remains completely unclear. Disciplines are significant drivers of new degree programs in digitalization because it is at the level of departments and faculties that program innovation happens, and not at the level of universities. A recent example is the rapid diffusion of data science study programs in universities around the globe.

In this situation, student numbers are gaining in importance. Digitalization is rapidly changing student and labor market demands (Börner et al., 2018; Frey & Osborne, 2017) and thus leading to increased demand for certain disciplines and degree programs while others lose importance. This shift has become more visible and urgent in universities because of the concurrent arrival of low-birth cohorts and the flattening of HE expansion (OECD, 2008; Vincent-Lancrin, 2020). This leads to a more dynamic environment in which disciplines, organized in departments within universities, strive to attract the remaining students. This is exacerbated by the worsening of university funding, partly because education spending is stagnating or declining in many European countries as a result of high national debt (Tandberg, 2010). As we show, digitalization significantly affects competitive relations among Swiss universities in teaching.

## Background

The Swiss higher education system (SHES) provides the empirical context for this study. The public SHES comprises four types of universities: federal institutes of technology (ETH), cantonal research universities (CRUs), universities of applied sciences (UAS), and universities of teacher education (UTE). These institutions are governed by the cantons in the case of the CRU, UAS, and UTE and at the federal level in the case of the ETH.^1^ Although they are autonomous public institutions by law, universities are subject to various external influences, in particular political actors at the cantonal and federal levels. ETH and CRUs can be considered the most autonomous scientific institutions because they strive for excellence in basic research and are embedded in international competition for status (Brunsson & Wedlin, 2021; Musselin, 2018). As the largest and oldest institutions, they constitute the backbone of the highly internationalized SHES. By contrast, UAS are more focused on applied research, cooperation with regional economic stakeholders such as SMEs, and local labor markets (Weber et al., 2010). UTE enjoy the least autonomy because they are often more directly steered by cantonal HE politics (Eckert, 2019) to train the future regional education workforce.

However, despite the higher autonomy of CRUs and ETH from political demands, both types of universities are closely coupled with labor market demands. One reason is the historically high importance of the strong Swiss vocational education and training system (Wettstein et al., 2017), which accounts for around 60% of all students at secondary II level (ISCED-3), whereas university-track high schools account for around 30% of students (SKBF, 2023, p. 113). This has led to a comparatively low proportion of academics in the Swiss population (OECD, 2008, p. 167). Consequently, CRU and ETH graduates are generally well positioned to enter the labor market, resulting in very low graduate unemployment (SKBF, 2023, p. 246). Conversely, CRUs and ETH need to keep the employability of their graduates high for them to remain competitive with other universities, especially those from UAS. Rising unemployment due to lack of adaptability to changing labor market needs would have a negative impact on the legitimacy of universities in the eyes of politicians and the public. Therefore, Swiss universities pay close attention to changing labor market demands due to digitalization (Tratschin et al., 2023). Additionally, the free choice of university with a Swiss baccalaureate means that the reputation of a university is less important except for international students (SKBF, 2023, p. 233f.). Choosing a university by its geography plays a minor role due to the small size of the country and the excellent public transportation system (Denzler & Wolter, 2010).^2^ As a result, students are highly mobile and can easily choose and transfer between universities. This, in turn, increases competition between programs and disciplines to attract more students and thus access more public funding.

The SHES is a low-tuition, low-subsidy type of HE system (Garritzmann, 2023). It is primarily funded by the state and federal governments: 80% of the university budget is financed by the Confederation or the cantons (SBFI, 2024; SKBF, 2023). Only 20% is raised through competitive third-party funding, mostly also financed by the federal state (e.g., Swiss National Science Foundation grants) or European funding programs or through private donors such as commissioned research, enterprises, foundations, or philanthropists.^3^ At CRUs, 70% of federal is determined by student enrollment and degrees, effectively proxies for teaching performance, and 30% by research performance, mainly through competitive third-party funding (SBFI, 2024). At UAS, as much as 85% of federal funding is allocated by student enrollment and degrees, effectively proxies for teaching performance, and only 15% by research. The cantonal subsidies for both CRUs and UAS include contributions from other cantons that are 100% linked to student numbers because they compensate a cantonal university if it educates students from other cantons (EDK, 2024). Most cantonal contributions are linked to student numbers and degrees because politicians consider these to be the key performance indicators of universities and are easy to measure (see e.g. Kanton Luzern, 2022). Overall, student enrollment accounts directly or indirectly for approximately three quarters of the global budget of Swiss universities. Attracting more students is thus crucial to public legitimization and financial resources.

Previous studies have highlighted that the SHES legally and institutionally involves a dual approach of cooperation and competition (Baschung et al., 2009). At the organizational level, there are many examples of cooperation between universities in various thematic fields, for example through common research centers, dual professorships, and jointly offered degree programs. One example is the Digitalization Initiative of the Zurich universities.^4^ The initiative, which includes joint research clusters, innovation programs, and dual professorships, is funded by the Canton of Zurich with CHF 300 million over ten years (2020–2029) and intended to enhance and deepen collaboration between these universities in digitalization-relevant topics. At the same time, elements of competition have been integrated into institutional governance through PBF allocation (SBFI, 2024). Nonetheless, current HE policy encourages universities to define their positions through mutual agreements, a process described as soft or “tacit” competition (Tratschin et al., 2023).

However, there is one domain in which competition among Swiss universities has intensified recently, and that is teaching. Since the introduction of the UAS, the SHES has been diversified, and multiple study fields are offered by more than one university type, including business administration, computer science, and engineering (Weber et al., 2010). In these disciplines, competition has intensified between CRUs, ETH, and UAS for the local student population (Rostetter, 2020; Schwarzenbach, 2024). As a result of PBF allocation by student numbers and changing student demand*,* the recruitment of students, especially in digitalization-relevant programs, has become a central issue for Swiss universities.

**Fig. 1:**
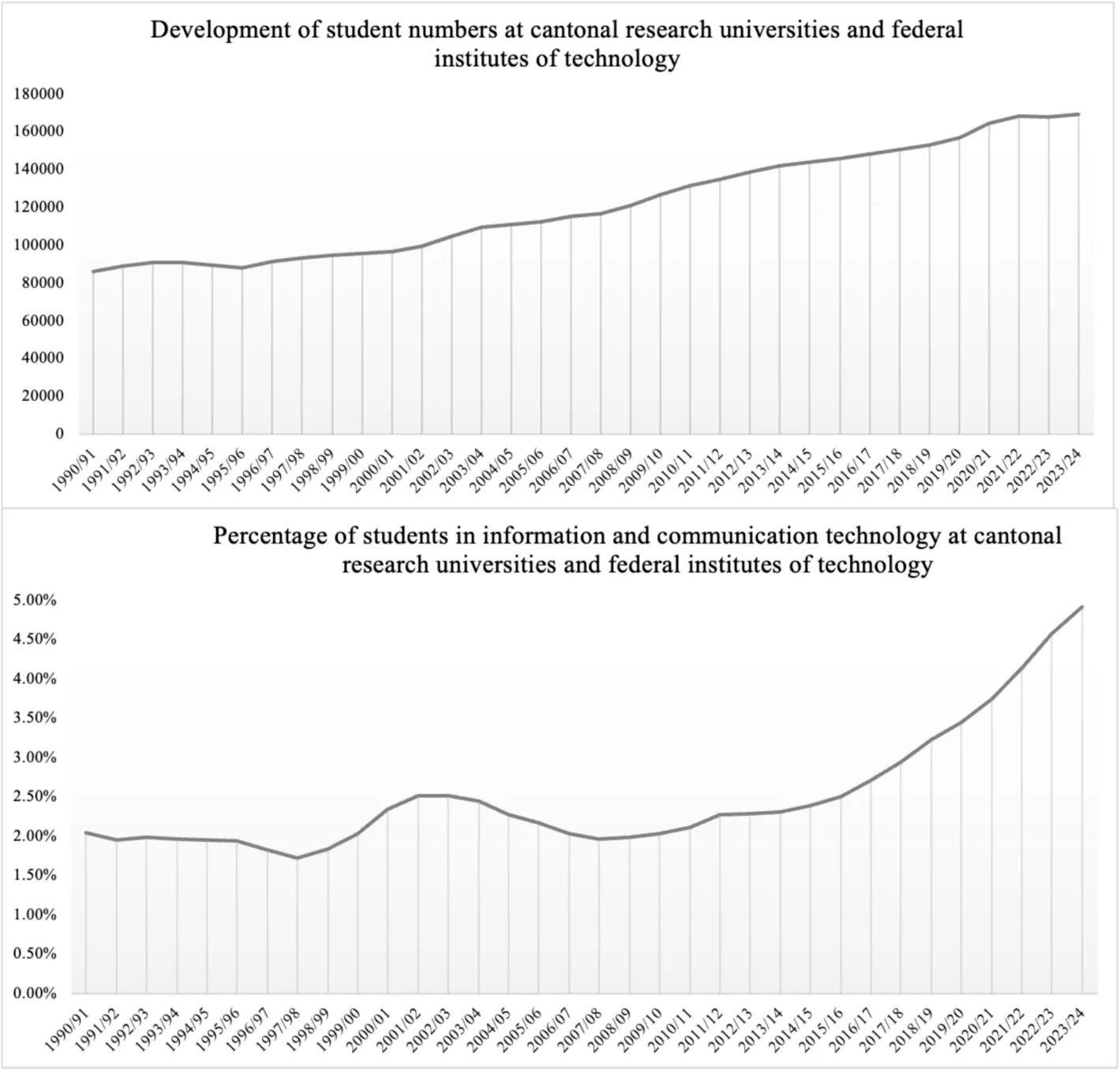
Development of student numbers and students in information and communication technology at cantonal research universities and federal institutes of technology. Source: BFS (2024)

Figure 1 illustrates changing student demands and the arrival of low-birth cohorts through declining student growth rates for CRUs and ETH from 1990 to 2024. The upper panel shows that student numbers in Switzerland have doubled since 1990. However, student numbers have stagnated since 2021, and these figures are expected to grow only moderately in the coming years (with annual growth rates of 1–2% until 2033 (BFS, 2025). The lower panel shows that the proportion of students in study programs addressing digitalization, approximated here by the proportion of programs on information and communication technologies,^5^ has increased rapidly since around 2016 and almost tripled since 1990. The student numbers in these fields are expected to grow by an additional 60% by 2033 (BFS, 2025). Considerable growth is also expected in the related disciplines of mathematics and physics.

Figure 1 suggests that scientific disciplines that link their field of study to emerging demands in digitalization will maintain or increase student numbers. Student numbers are thus gaining a new political dimension (Ammann et al., 2018; Rostetter, 2020). For example, the rapidly rising student numbers at ETH Zurich are compared with those stagnating at the University of Zurich (UZH) (Schwarzenbach, 2024). This is exacerbated by the stagnation of research and HE funding at the federal level as a result of rising budget deficits (Schatz, 2024). The basic tenor of this discussion is the continued high level of investments in disciplines that are no longer in demand. Universities are responding to these dynamics, as recent examples from the SHES show: as part of the UZH’s Digital Society Initiative, 36 additional professorships “dealing with digital transformation-relevant issues” have been established (von Däniken, 2023; our translation). However, only one third of these positions were introduced in technical disciplines such as computer and data sciences. Another example includes the Zug Institute for Blockchain Research at the University of Lucerne, financed by the Canton of Zug. The new institute includes nine additional professorships involved in blockchain research in law, economics, philosophy, medicine, and health sciences (Fulterer, 2024). Both cases are insightful because the majority of investments are not in technical disciplines but in social sciences and medicine with a focus on digital issues.

## Methodology

Here, we test empirically whether and, if so, at which levels competition for students in Swiss HE occurs, using digitalization study programs as a case study. Our analysis examines the extent to which these study programs offered by a discipline within a university depend on programs offered by the same discipline in other universities, by other disciplines within the same university, and within the same cultural or geographical region.

### Data and measurements

We use a database of study programs (Leder et al. 2024) at bachelor and master, doctoral, and further education (certificate, diploma, and masters of advanced studies) levels that relate to digitalization offered by all Swiss universities between 2017 and 2023.^6^ The data was collected through a query search of program catalogs for each university using English-language key terms related to technological aspects of digitalization and their equivalents in German, French, and Italian.^7^ Next, the study program descriptions were used to evaluate whether the programs indeed deal with digitalization. A total of 927 study programs on digitalization at 35 universities were in place or introduced during this period. This accounts for 21% of all degree programs offered at all Swiss universities.

**Fig. 2:**
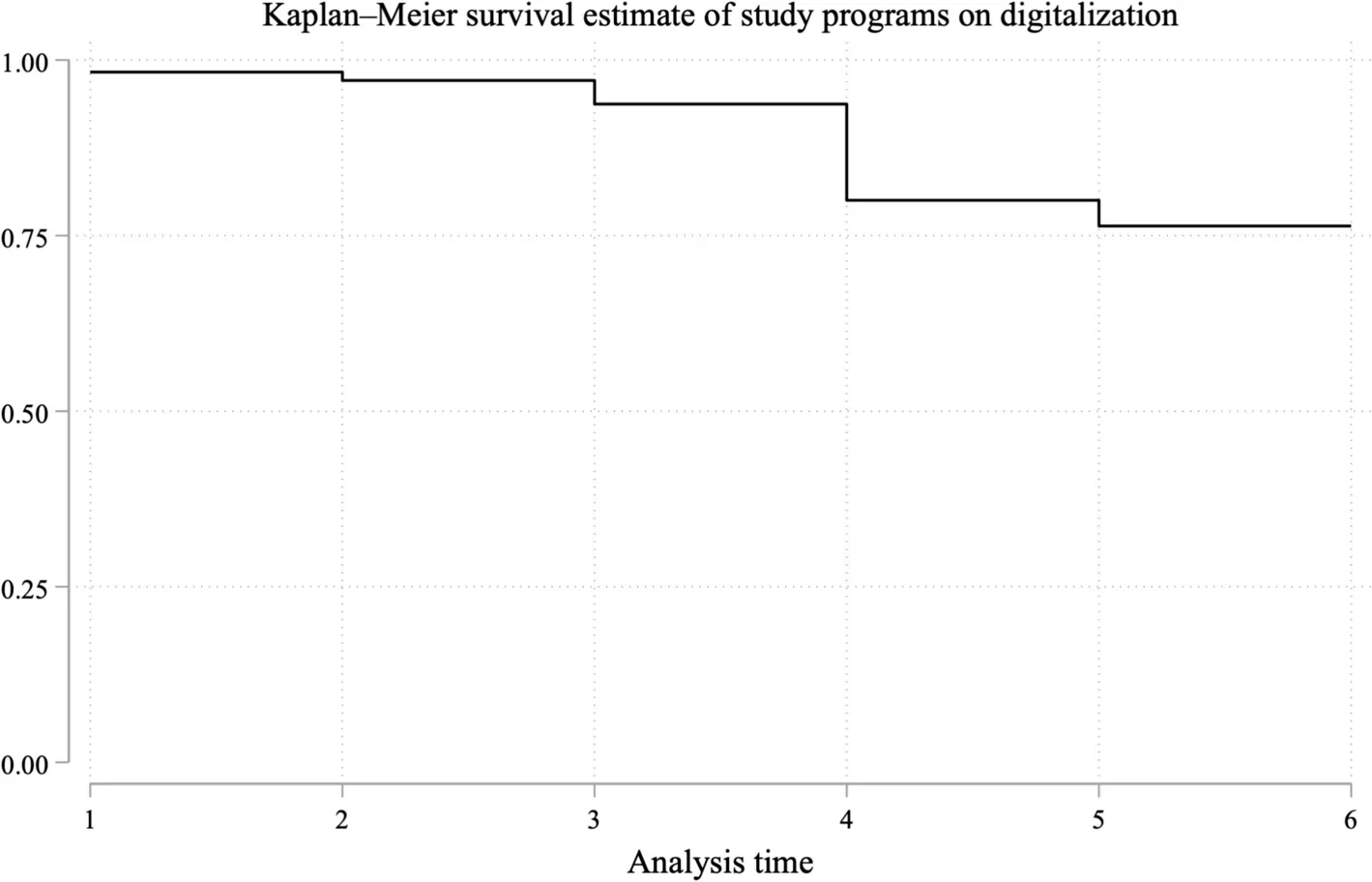
Mortality of new study programs on digitalization of a discipline at each university in the observed time period between 2017–2023. Source: Data on digitalization study programs of Swiss universities 2017–2023. Note: The figure looks at the survival of study programs from their foundation

The dataset includes both new programs that did not previously exist, such as in data science, and existing programs that were rebranded, for example from marketing to digital marketing. Many of these programs certainly use a lot of buzz to market their offerings and attract new students. However, the growing student demand for digitalization-related programs signals a substantive change in the SHES (see Fig. 1). In addition, there is a certain degree of program mortality in our dataset: As Fig. 2 shows, about 25% of the programs did not survive the study period.

The 35 universities offering study programs on digitalization between 2017 and 2023 cover all four types and are spread across 17 cantons and German, French, and Italian language regions. Disciplines were grouped according to the classification by the Swiss Federal Statistical Office. These include (1) architecture, construction, and planning, (2) educational science and teacher training, (3) humanities, art, and design, (4) medicine and health, (5) natural sciences and technology, (6) social sciences, law, and social work, and (7) economy and services. Although this classification of disciplinary groupings is rather broad, it enables us to establish comparability between university types. On average, each university hosts 3.1 disciplines offering study programs on digitalization, ranging from just one grouping to all seven.

Table 1 provides an overview of the nested structure of the dataset. Overall, we observe 109 study programs on digitalization located in the 35 universities. Most of the programs are offered in economy and services (23 universities) and in the natural sciences and technology (21 universities) and the fewest programs in architecture, construction, and planning (7 universities). Furthermore, most programs are offered in the German-speaking Switzerland (79 programs offered in 26 universities), in UAS (49 programs in 11 UAS) and CRU (41 programs in 11 CRU), and in the Canton of Zurich (24 programs in 7 universities).

**Table 1:** Nested structure of the data set

	(1)	(2)	(3)
VARIABLES	N yearly observations	N scientific disciplines	N universities
Scientific disciplines			
Architecture, construction and planning	49	7	7
Educational science and teacher training	133	19	19
Humanities, art and design	91	13	13
Medicine and health	84	12	12
Natural sciences and technology	147	21	21
Social sciences, law and social work	98	14	14
Economy and services	161	23	23
Linguistic and cultural regions			
German-speaking Switzerland	553	79	26
French-speaking Switzerland	147	21	7
Italian-speaking Switzerland	63	9	2
University types			
Federal institutes of technology (ETH)	49	7	2
Cantonal research universities (CRUs)	308	41	11
Universities of applied sciences (UAS)	329	49	11
Universities of teacher education (UTE)	91	12	11
Administrative regions (cantons)			
*AG (Argovia)*	*49*	*7*	*1*
-Universities of applied sciences (UAS)	49	7	1
*BE (Berne)*	*91*	*13*	*5*
-Cantonal research universities (CRUs)	28	4	1
-Universities of applied sciences (UAS)	42	6	1
-Universities of teacher education (UTE)	21	3	3
*BS (Basle-City)*	*35*	*5*	*1*
-Cantonal research universities (CRUs)	35	5	1
*FR (Fribourg)*	*14*	*2*	*1*
-Cantonal research universities (CRUs)	14	2	1
*GE (Geneva)*	*42*	*6*	*1*
-Cantonal research universities (CRUs)	42	6	1
*GR (Grisons)*	*42*	*6*	*2*
-Universities of applied sciences (UAS)	35	5	1
-Universities of teacher education (UTE)	7	1	1
*JU (Jura)*	*28*	*4*	*1*
-Universities of applied sciences (UAS)	28	7	1
*LU (Lucerne)*	*70*	*10*	*3*
-Cantonal research universities (CRUs)	21	3	1
-Universities of applied sciences (UAS)	42	6	1
-Universities of teacher education (UTE)	7	1	1
*NE (Neuchâtel)*	*14*	*2*	*1*
-Cantonal research universities (CRUs)	14	2	1
*SG (St Gall)*	*56*	*8*	*3*
-Cantonal research universities (CRUs)	28	4	1
-Universities of applied sciences (UAS)	14	2	1
-Universities of teacher education (UTE)	28	4	1
*SO (Solothurn)*	*7*	*1*	*1*
-Universities of teacher education (UTE)	7	1	1
*SZ (Schwyz)*	*7*	*1*	*1*
-Universities of teacher education (UTE)	7	1	1
*TG (Thurgovia)*	*7*	*1*	*1*
-Universities of teacher education (UTE)	7	1	1
*TI (Ticino)*	*63*	*9*	*2*
-Cantonal research universities (CRUs)	42	6	1
-Universities of applied sciences (UAS)	21	3	1
*VD (Vaud)*	*49*	*7*	*2*
-Federal institutes of technology (ETH)	21	3	1
-Cantonal research universities (CRUs)	28	4	1
*VS (Valais)*	*21*	*3*	*2*
-Cantonal research universities (CRUs)	14	2	1
-Universities of applied sciences (UAS)	7	1	1
*ZH (Zurich)*	*168*	*24*	*7*
-Federal institutes of technology (ETH)	28	4	1
-Cantonal research universities (CRUs)	42	6	1
-Universities of applied sciences (UAS)	91	13	4
-Universities of teacher education (UTE)	7	1	1

Our dependent variable is the yearly number of study programs on digitalization in a discipline at each university. Our main independent variables are the levels at which competition for students can take place: the university, canton, linguistic region, university type, and discipline. These levels represent fields in which actors’ relationships center on scarce resources, either because the university management or the canton distributes the global budget among the participants according to student numbers or because the number of students potentially available within a university type, language region, or discipline is limited. For each level, we measure the yearly number of study programs on digitalization. We assume that competition accompanies mutual observation and that, consequently, an increase in competitors’ supply results in an increase in supply by a discipline within a university.

We introduce several control variables that correlate with the number of programs offered and the amount of competition. We control for year, canton, linguistic region, university type, and discipline with dummies. For each year, we measure whether the university has introduced a digitalization strategy.^8^ In 2017, only 4 of the 35 universities had a digitalization strategy. By 2023, this figure had risen to 22. This steep increase in the adoption of digitalization strategies can be seen as an effect of mimetic isomorphism (DiMaggio & Powell, 1983) in the organizational field of Swiss universities. At the university level, we also control for the yearly number of students and the percentage of revenue from continuing education. Finally, we add control variables for the programs on digitalization. At the level of disciplines located in the universities, we (1) count the total number of ECTS awarded, correcting for the number of study programs; (2) count the proportion of programs dedicated to continuing education, a state-independent source of income from student numbers; and (3) approximate the uniqueness of the programs^9^ from the study program description to control for competitive advantages. Table 2 lists the descriptive statistics of all variables.

**Table 2:** Descriptive statistics

	(1)	(2)	(3)	(4)	(5)
VARIABLES	*N* observations (*n* scientific disciplines nested in *n* universities)	mean	sd	min	max
Dependent variable					
*N* study programs on digitalization of a scientific discipline at each university	763 (109/35)	4.865	7.493	0	47
Competition					
*N* study programs on digitalization within the university	763 (109/35)	23.65	20.89	0	81
*N* study programs on digitalization within the administrative region (cantons)	763 (109/35)	66.53	68.40	0	241
*N* study programs on digitalization within the linguistic/cultural region	763 (109/35)	319.4	202.5	22	607
*N* study programs on digitalization within the university type	763 (109/35)	209.4	147.7	8	494
*N* study programs on digitalization within the scientific discipline	763 (109/35)	99.22	104.5	3	357
Digitalization strategies introduced					
Digitalization strategy of the university	763 (109/35)	0.491	0.500	0	1
Further control variables					
Number of students per university	763 (109/35)	10,957	7,777	332	28,988
Percentage of revenue from continuing education per university	763 (109/35)	0.0520	0.0435	0	0.185
Uniqueness of the course based on the self-description	763 (109/35)	0.320	0.0904	0.0511	0.613
Total ECTS awarded	763 (109/35)	57.42	48.91	0	180
Proportion of continuing education programs on digitalization	763 (109/35)	0.642	0.369	0	1

## Method

Our data comprises a strongly balanced panel dataset grouped around the discipline at each university and year. The analytical units are 109 disciplines (at department or faculty level) located in 35 universities, consisting of 763 observations between 2017 and 2023. We use random-effect regression models to include nonvarying control variables such as canton and university type. Nevertheless, the results are robust in fixed-effect regression models; the main effects remain significant and equal in effect size. We present three predictions. First, we predict the correlation between the independent variables and the dependent variable. Because competition has a time-delayed effect, we also calculate models that consider time lags in the dependent variable of one and two years. These involve fewer observations, so the robustness of the findings increases with time delay but decreases with the number of observations.

## Results

### Descriptive findings

**Fig. 3:**
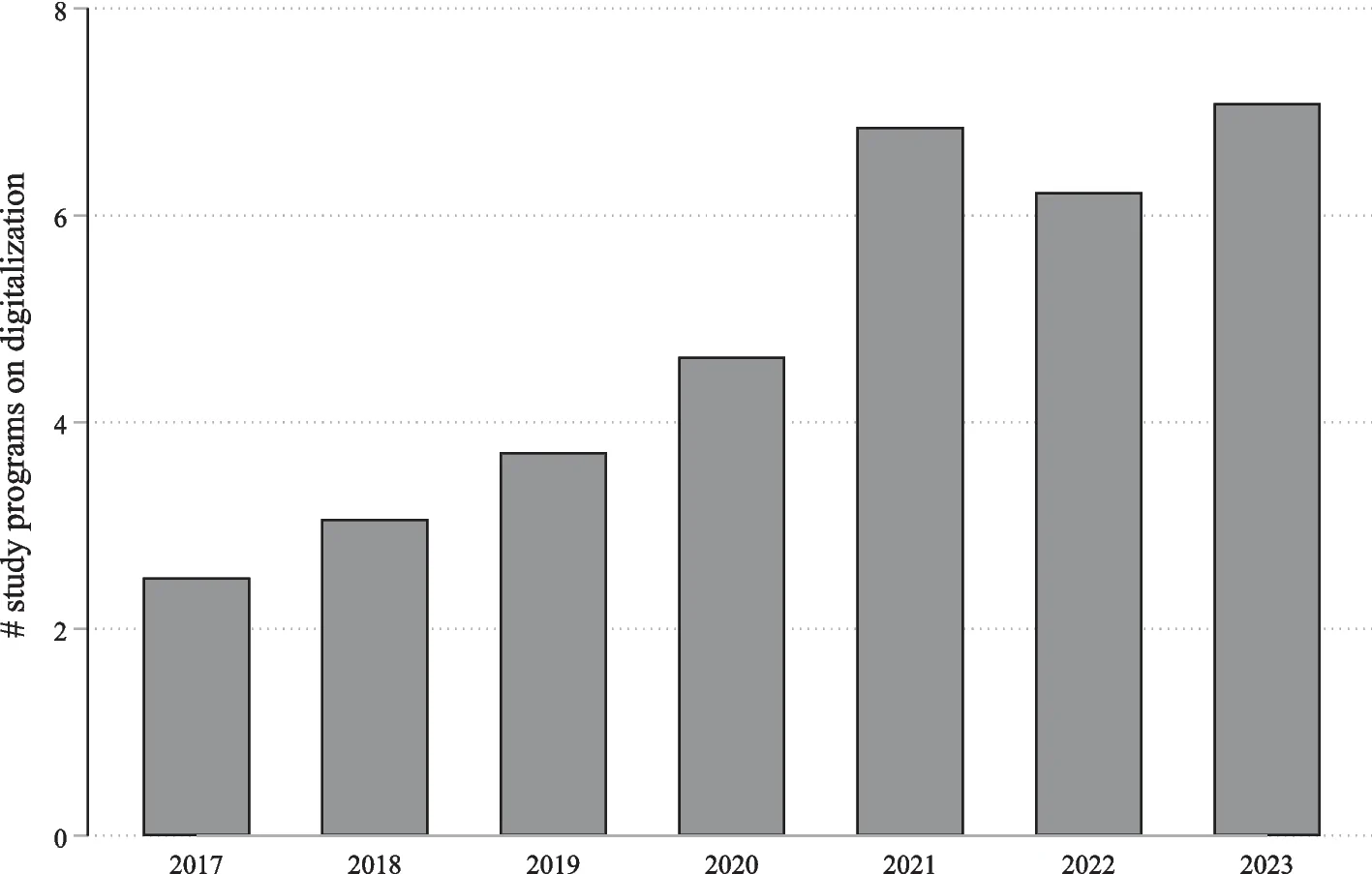
Development of digitalization study programs of a discipline at each university. Source: Data on digitalization study programs of Swiss universities 2017–2023

Figure 3 illustrates the yearly development of study programs offered on digitalization within the disciplines at all universities. The figure shows that supply more than tripled during the seven-year observation period. Such an increase can be observed across cantons, language regions, university types, and disciplines. However, the temporary decline in 2022 also implies that not all programs have survived due to the dynamic situation in this program segment (see also Figure 2).

**Fig. 4:**
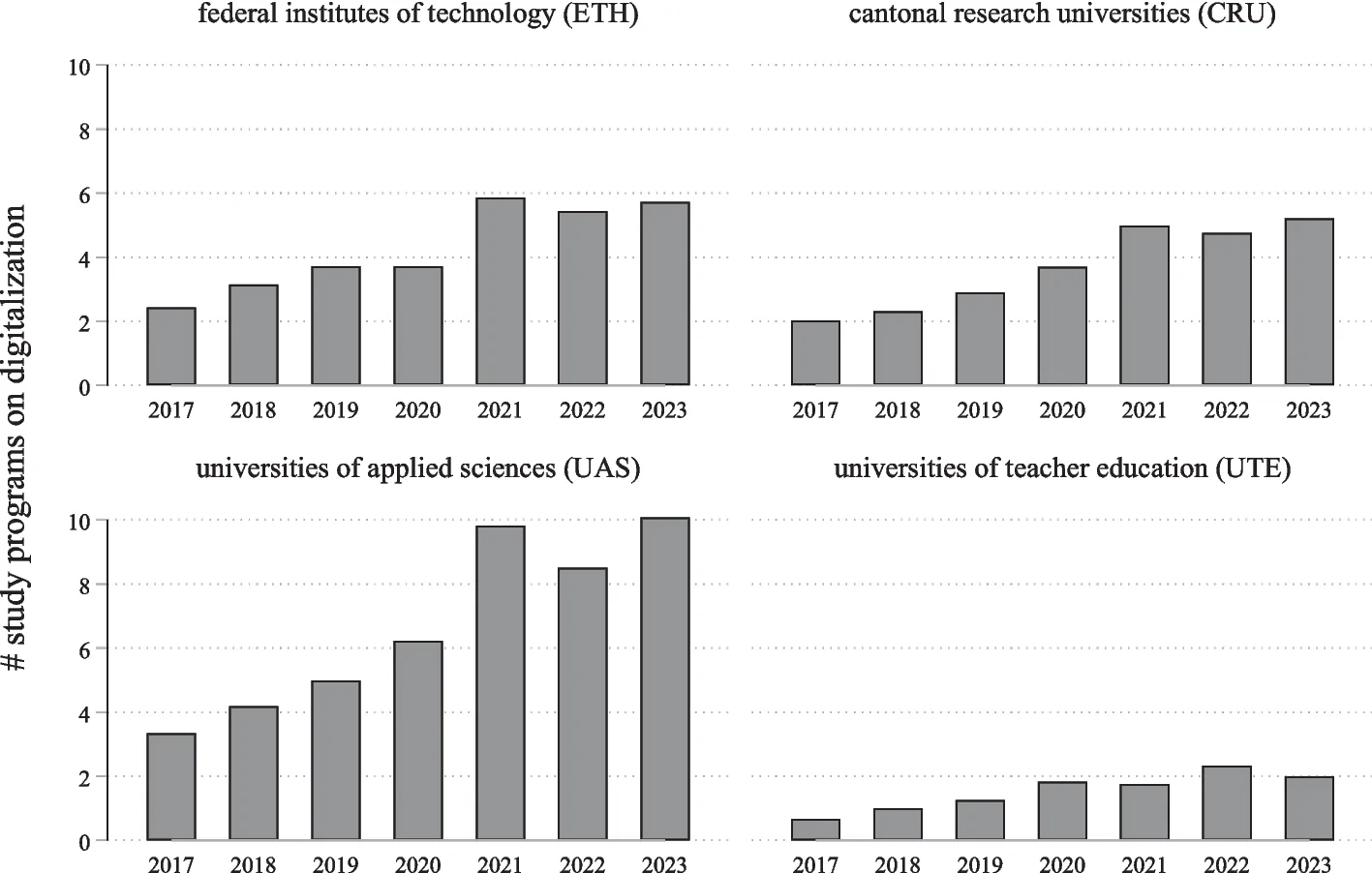
Development of digitalization study programs of a discipline at each university among the university types. Source: Data on digitalization study programs of Swiss universities 2017–2023

Figure 4 differentiates the types of university and shows that the range of digitalization-related programs is increasing across all types. The largest growth in programs is an average of 10 programs per institution in 2023 at UAS, which outpaces all other university types. CRUs and ETH, each with an average of 5–6 programs per institution, show similar growth rates and numbers of programs per institution. However, UAS appear to react faster and more flexibly to student demand. UTE react most sluggishly, with significantly lower levels than the other types: an average of 2 programs at each UTE.

**Fig. 5:**
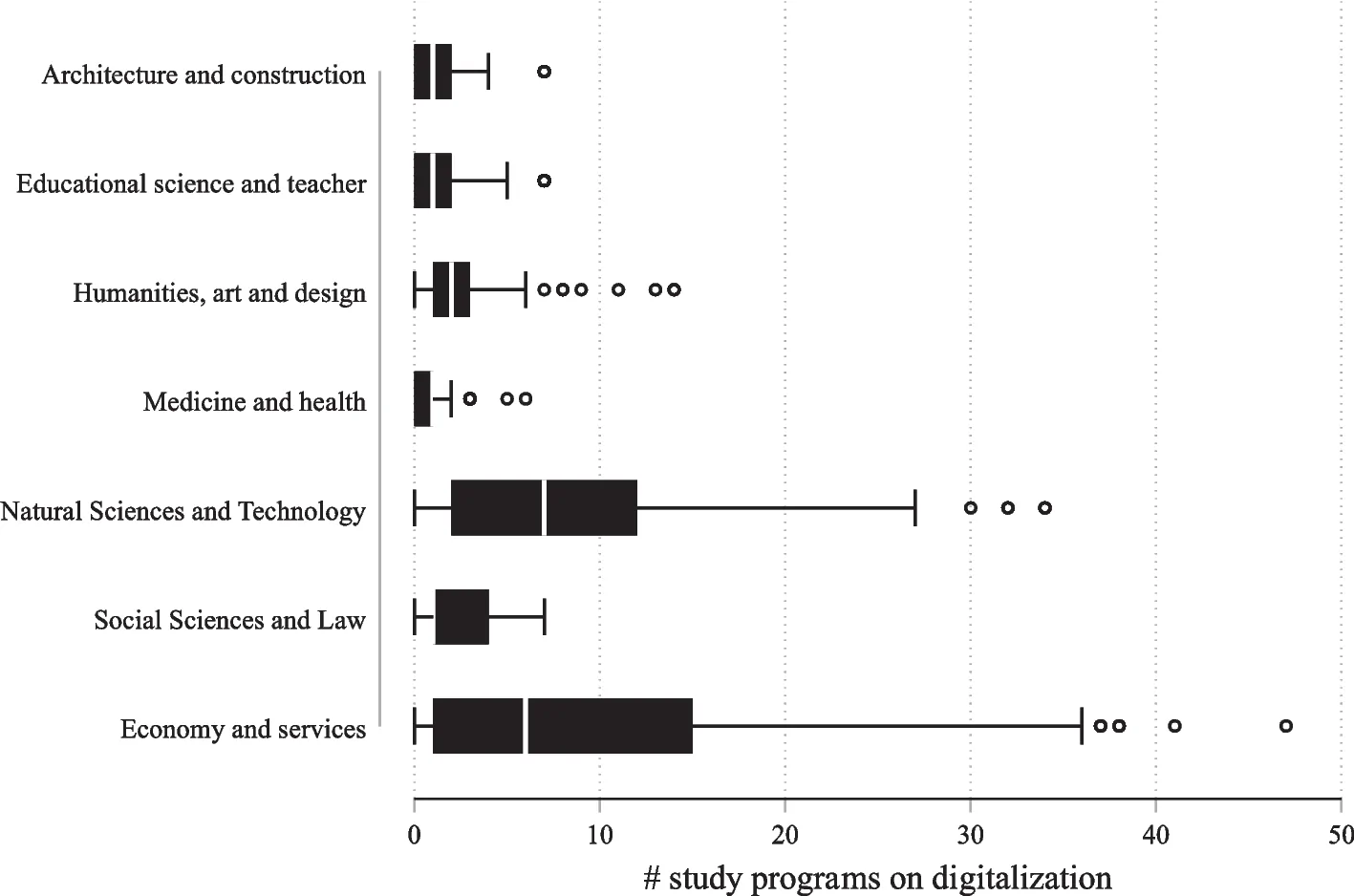
Number of digitalization study programs within the disciplines at each university. Source: Data on digitalization study programs of Swiss universities 2017–2023

Figure 5 shows how the number of digitalization study programs varies within the disciplines. The economy and services and natural sciences and technology groupings offer digitalization-related programs at the most universities and the highest number of programs. Nevertheless, the numbers of programs offered within individual disciplines at different universities and in different years vary considerably. Next, we seek to explain this variance by analyzing whether competition can be observed and where it occurs.

### Multivariate findings

**Fig. 6:**
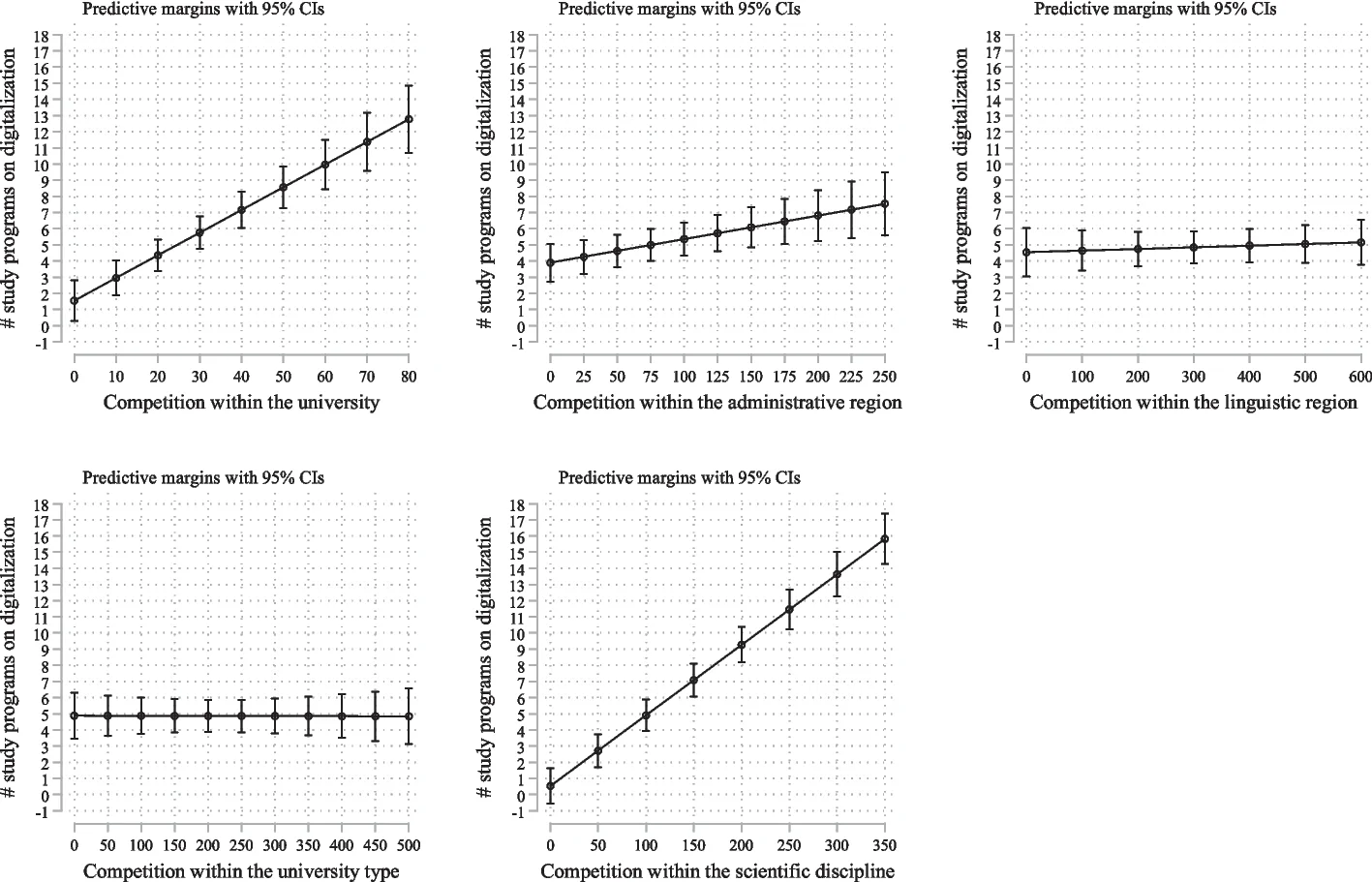
Competition as a driver of the number of digitalization study programs of a discipline at each university. Source: Data on digitalization study programs of Swiss universities 2017–2023. Note: Results of Table 2, column 1

Table 3 documents the multivariate results. Figure 6 illustrates the main findings of column 1: the marginal effects of the yearly number of digitalization-related degree programs at the level of (1) university, (2) canton, (3) language region, (4) university type, (5) and discipline on the number of digitalization-related programs in a discipline at each university. We find three significant effects of competition: The higher the number of programs at a university, canton and discipline, the higher the number of programs in a discipline at each university. The effects are rather high. As shown by the coefficients in Table 3, column 1, the number of programs on digitalization offered in a discipline at a university increases (1) by an average of 1.4 programs if the number of digitalization programs offered at a university increases by 10 programs (0.140***), (2) by 0.4 programs if the number of digitalization programs offered in the discipline increases by 10 programs (0.0437***), and (3) by 0.15 programs if the number of digitalization programs offered at the canton increases by 10 programs (0.0146***). As illustrated in Fig. 6, the overall effect of the disciplines is as high as the effect of the university because the range of programs offered in the disciplines is greater. The competitive effect of the administrative region is comparatively small. However, these findings are static because they consider no reaction time to ongoing competition.

**Table 3:** Determinants of the yearly offer of digitalization study programs of a discipline at each university

	(1)	(2)	(3)
VARIABLES	*N* study programs on digitalization of a scientific discipline at each university	*N* study programs on digitalization of a scientific discipline at each university (1lag)	*N* study programs on digitalization of a scientific discipline at each university (2lags)
Competition			
*N* study programs within the university	0.140***	−0.000444	−0.00489
	(0.00232)	(−1.09e-05)	(−0.000134)
*N* study programs within the administrative region (cantons)	0.0146***	0.0128**	0.00507
	(6.82e-05)	(7.98e-05)	(3.53e-05)
*N* study programs within the linguistic/cultural region	0.00102	0.000809	0.00268
	(1.80e-06)	(2.04e-06)	(7.12e-06)
*N* study programs within the university type	−7.71e-05	0.00834**	0.00712*
	(−1.90e-07)	(2.91e-05)	(2.74e-05)
*N* study programs within the scientific discipline	0.0437***	0.0254***	0.0193***
	(0.000108)	(8.74e-05)	(6.99e-05)
Digitalization strategies introduced			
Digitalization strategy of the university	−0.871***	−0.293	−0.410
	(−0.281)	(−0.125)	(−0.181)
Years			
2017 (reference group)			
2018	−0.203	0.140	0.534
	(−0.0677)	(0.0559)	(0.205)
2019	−0.769**	0.364	2.168***
	(−0.284)	(0.166)	(0.975)
2020	−1.231***	1.722***	0.827
	(−0.576)	(1.036)	(0.505)
2021	−3.035***	−1.284	−0.285
	(−2.154)	(−1.237)	(−0.284)
2022	−2.407***	0.288	
	(−1.516)	(0.242)	
2023	−3.053***		
	(−2.273)		
Administrative regions (cantons)			
AG (reference group)			
BE	−0.129	2.554	3.941
	(−0.367)	(9.077)	(15.08)
BS	0.402	4.196	5.712
	(1.506)	(19.60)	(28.59)
FR	−3.560	−2.035	−1.170
	(−17.24)	(−12.25)	(−7.539)
GE	−1.599	2.827	3.912
	(−9.512)	(20.88)	(30.89)
GR	−1.661	−4.523	−3.787
	(−5.411)	(−18.38)	(−16.51)
JU	−0.685	3.039	3.071
	(−4.092)	(22.53)	(24.35)
LU	0.262	1.873	3.284
	(0.802)	(7.167)	(13.58)
NE	−6.613	1.518	3.795
	(−47.16)	(13.48)	(36.12)
SG	−3.565	−1.213	−0.321
	(−12.37)	(−5.252)	(−1.493)
SO	−2.009	−5.951	−5.517
	(−13.55)	(−49.87)	(−49.44)
SZ	1.146	1.906	2.861
	(7.200)	(14.88)	(23.90)
TG	−0.255	1.497	2.218
	(−1.595)	(11.66)	(18.48)
TI	-	-	-
VD	−3.091	2.693	4.475
	(−18.80)	(20.36)	(36.21)
VS	−5.024	1.915	3.926
	(−23.35)	(11.11)	(24.48)
ZH	0.158	3.999	5.923*
	(0.419)	(13.15)	(20.81)
Linguistic regions			
German-speaking Switzerland (reference group)			
French-speaking Switzerland	1.937	0.160	0.227
	(9.149)	(0.940)	(1.424)
Italian-speaking Switzerland	−1.668	2.346	4.323
	(−5.332)	(9.416)	(18.71)
University types			
Federal institutes of technology (ETH) (reference group)			
Cantonal research universities (CRUs)	−0.226	−0.925	−0.456
	(−0.660)	(−3.362)	(−1.769)
Universities of applied sciences (UAS)	−1.208	−1.480	0.228
	(−3.453)	(−5.272)	(0.862)
Universities of teacher education (UTE)	1.627	−2.133	−0.959
	(6.602)	(−10.75)	(−5.161)
Scientific disciplines			
Architecture, construction and planning (reference group)			
Educational science and teacher training	0.956	3.284	3.855
	(2.683)	(11.44)	(14.35)
Humanities, art and design	2.756	4.419	4.677
	(7.497)	(14.92)	(16.88)
Medicine and health	0.560	2.764	2.916
	(1.509)	(9.253)	(10.44)
Natural sciences and technology	3.965	5.864*	7.632**
	(10.01)	(18.40)	(25.54)
Social sciences, law and social work	3.172	3.609	4.118
	(8.328)	(11.76)	(14.35)
Economy and services	2.770	7.116**	9.771***
	(6.948)	(22.18)	(32.42)
Further control variables			
Number of students per university	−9.71e-05	−0.000192	−0.000110
	(−1.17e-08)	(−2.97e-08)	(−1.87e-08)
Percentage of revenue from continuing education per university	11.73	52.23*	53.39*
	(253.5)	(1,402)	(1,531)
Uniqueness of the course based on the self-description	−0.111	3.689	3.524
	(−0.720)	(29.69)	(30.31)
Total ECTS awarded	0.0113	−0.0211	−0.0158
	(0.000292)	(−0.000681)	(−0.000546)
Proportion of continuing education programs on digitalization	3.833	0.482	1.202
	(14.33)	(2.241)	(5.972)
Constant	0.0146***	0.0128**	0.00507
	(6.82e-05)	(7.98e-05)	(3.53e-05)
Number of observations	763	654	545
Number of scientific disciplines at each university	109	109	109

**Fig. 7:**
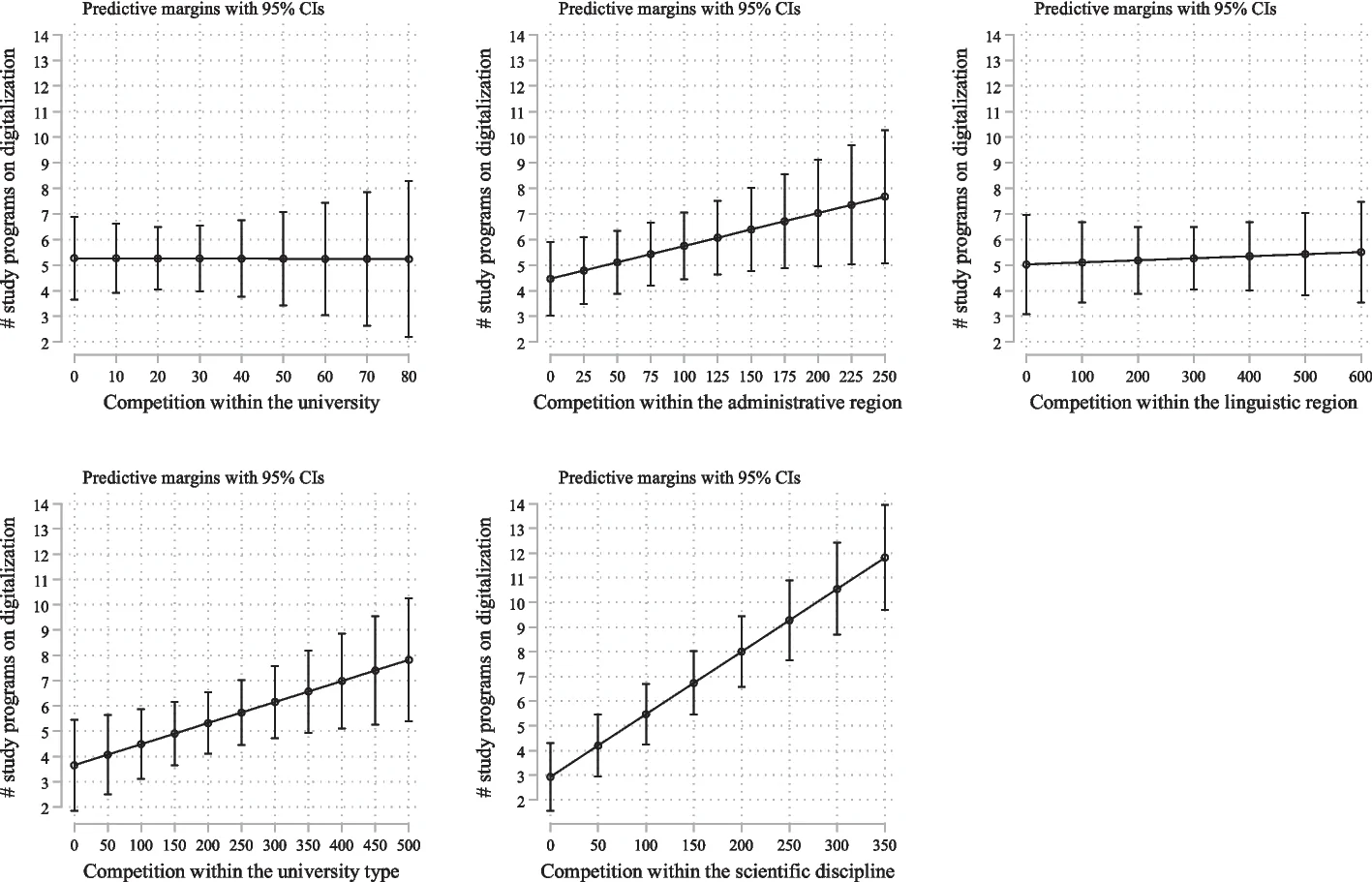
Competition as a driver of the number of digitalization study programs of a discipline at each university (1 year time-delayed effect) Source: Data on digitalization study programs of Swiss universities 2017–2023. Note: Results of Table 2, column 2

Figure 7 illustrates the main findings of Table 3, column 2, considering a time delay of competition of one year. Only the effects of the total study program offering at the level of the canton and the discipline remain significant. Furthermore, the total study program offering at the level of the university type becomes significant. However, the strongest relative and absolute effects can be observed for competition in disciplines: if the number of digitalization programs offered in a discipline increases by 10 programs, the number of programs on digitalization offered in this discipline at a specific university increases in the next year by an average of 0.25 programs (0.0254***). In contrast, if the number of digitalization programs offered in a canton increases by 10 programs, the number of programs on digitalization offered in a discipline at a specific university increases in the next year by an average of 0.13 programs (0.0128**). The number of such programs increases by 0.8 if the number of digitalization programs offered in a university type increases by 10 programs (0.00834**). As Fig. 7 shows, the absolute effect of discipline is the greatest by far.

**Fig. 8:**
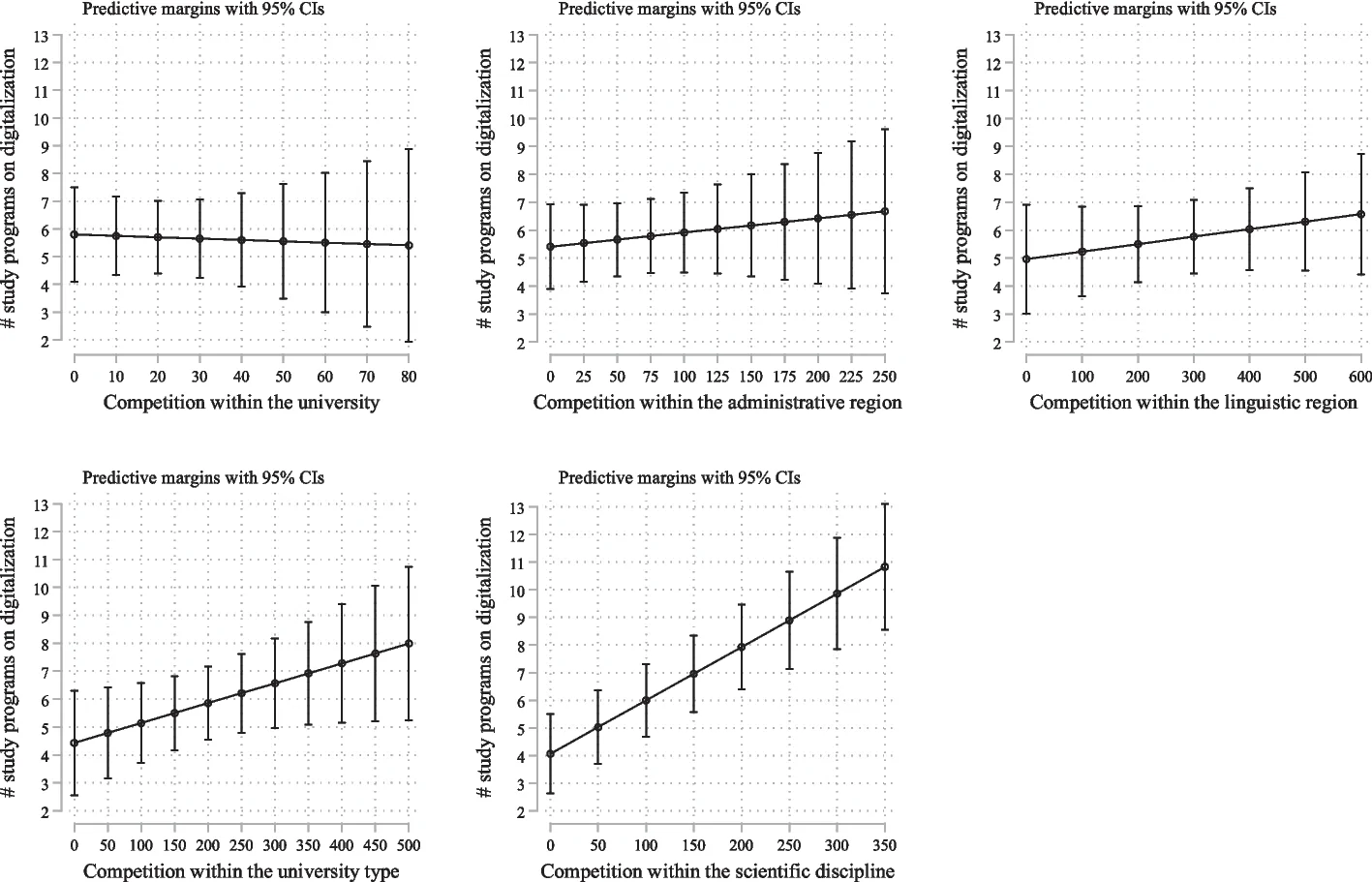
Competition as a driver of the number of digitalization study programs of a discipline at each university (2-year time-delayed effect). Source: Data on digitalization study programs of Swiss universities 2017–2023. Note: Results of Table 2, column 3

Figure 8 considers a time delay of ongoing competition of two years (see Table 3, column 3). Only two effects remain significant: competition in the discipline and the university type. Again, the strongest and most significant effect can be observed in discipline: if the number of digitalization programs offered in a discipline increases by 10 programs, the number of programs on digitalization offered in this discipline at a specific university increases in the next two years by an average of 0.19 programs (0.0193***). The effect of the university type remains significant only at the 10% level: if the number of digitalization programs offered in a university type overall increases by 10 programs, the number of programs on digitalization offered in a discipline at a specific university of this type increases in the next two years by an average of 0.07 programs (0.00712*). Overall, the results confirm our prediction that competition for students is strongest within disciplines. We also find weaker forms of competition in university, canton and university type that correspond with our reflections on the distribution of the global budget. As a robustness check, we rerun the regression analyses by clustering standard errors according to the 35 universities. The effects remain stable and significant.

The analyses confirm that the range of programs on digitalization has increased significantly, that economy and services and natural sciences and technology offer the most programs, and that universities that generate a large proportion of their income from continuing education offer more programs on digitalization. These results are consistent with previous observations and theoretical considerations. Interestingly, the introduction of a digital strategy is significantly negatively correlated with the digitalization-relevant study program offerings at a university in the short term but shows no effects after 1–2 years. One possible interpretation is that universities with fewer programs than their direct competitors see a more urgent need to introduce such a strategy. Because this effect disappears after 1–2 years, a university may gain some success in implementing a digital strategy, which would support this interpretation.

## Discussion and conclusion

In this paper, we asked whether there is competition between Swiss universities in academic teaching in the context of digital transformation and, if so, at what level this competition takes place. Our empirical analysis of a dataset of study programs on digitalization offered by Swiss universities between 2017 and 2023 shows a striking increase in such programs across university types, disciplines, and administrative and linguistic regions. The wide diffusion of digitalization study programs signals their attractiveness and relevance to students and legitimacy to external stakeholders, especially political and economic actors, who expect universities to address major societal challenges and provide a skilled workforce. For universities, digitalization study programs are a lucrative growth area for attracting more students and, mediated by PBF allocation, for obtaining more resources. Consequently, the rapid rise of these degree programs, the changing student demand for digitalization, and the mortality of a certain number of these programs all indicate a new dynamic in the SHES, which is otherwise considered a rather stable field (Baschung et al., 2009; Tratschin et al., 2023). We interpret this new dynamic as an effect of intensified competition for students among Swiss universities.

The multivariate analyses confirm that competition for students occurs mainly within disciplines. Disciplines, organized into university departments, have direct financial incentives to attract more students through program innovation in their domains. Competition is particularly intense in those disciplines where study programs are offered at various types of universities and seek to attract the same pool of qualified students: business administration, computer science, and engineering. Given the moderate growth in student enrollment expected in the coming years due to demographic changes and stagnating public investment in HE, we expect competition for students in teaching to intensify.

We also observe competition between university types, albeit at a more moderate level. This aligns with an institutional perspective that examines integration and profile building within the SHES (Tratschin et al., 2023; Weber et al., 2010): The introduction of UAS has not only diversified the SHES but also intensified competition between university types, particularly between CRUs and UAS, and to a lesser extent with ETH as well. However, as Tratschin et al. (2023) have argued, this is rather a soft or “tacit competition” for attention and legitimacy within the public and political sphere.

We observe significant differences between university types in changes among disciplines, particularly between CRUs and UAS. The number of programs at CRUs is also growing overall, but less than at UAS and ETH. CRUs are less invested in technical sciences and continuing education than are UAS and ETH. They are particularly strong in humanities, social sciences, law, and medicine, which are effectively not offered at UAS or ETH. CRUs attract the majority of their students in these disciplines (BFS, 2025), but these disciplines offer far fewer digitalization programs. One reason could be the absorption of the topic within curricula, which we cannot observe with our dataset at the program level. Although CRUs certainly have strong economics and natural science departments, these cannot account for the same dynamic as their competitor departments at ETH and UAS. At ETH, the main drivers are the natural sciences and technology disciplines, considered to be the traditional core competency of these institutions, where most program innovation originates. These programs not only attract more mobile, high-status, and high-performance students from within Switzerland (Denzler & Wolter, 2010) but also a rapidly growing number of international students (Schwarzenbach, 2024).

UAS are proving to be remarkably agile, active, and to some extent entrepreneurial providers, particularly in the field of continuing education (Weber et al., 2010). This is a lucrative area for most universities, because it generates a large amount of revenue due to its higher tuition fees than bachelor, master, and doctoral HE education. UAS seem to be very quick in creating new programs and in adapting existing ones to new trends. In contrast, UTE seem more reluctant to invest in digitalization-related study programs. As small and monothematic institutions that are more directly steered by cantonal governments, they have less to fear from competition in digitalization. Another reason also seems plausible: UTE students are the least mobile student group in the SHES (Denzler & Wolter, 2010). This reduces the incentives for UTE to attract students from other geographical regions beyond their own.

This raises the question of the nestedness (Hasse & Krücken, 2013; Hüther & Krücken, 2016) of disciplines as fields within universities. At what level does competition for students, researchers, and research grants actually occur? Whereas much multilevel competition literature examines relations of competition at university level in regional, nation-state, or international comparison (Bloch, 2021; Bloch et al., 2024; Krücken, 2021; Musselin, 2018), our results suggest examining disciplines within universities. This resonates with recent work that considers disciplines to be more agentic than mere vessels for scholarly communication (Meier, 2023). Indeed, through PBF allocation in many European HE systems, disciplines are important collective actors within universities in securing the means of production and reproduction, mainly by attracting sufficient numbers of students to teach as a joint effort by a disciplinary department. Securing basic resources then also leads to better positioning in representation on decision-making bodies and establishing favorable rules of the game overall (Meier, 2023).

Disciplines that can offer programs that are considered relevant to the labor market, such as digitalization and AI, have a better chance of long-term survival in their universities. Consequently, universities might specialize in certain disciplinary fields instead of offering the entire range of disciplines as they have so far. Increasing competition between types of universities is leading to a new segmentation in these positional markets (Ingram & Roberts, 2000; White, 1981). This new segmentation could result in smaller, declining disciplines being represented at fewer universities. This idea has already been the subject of political discussion, for example in Switzerland (Ammann et al., 2018; Sporn & Aeberli, 2004), where it met with understandable resistance from academics and universities. Because growth rates for students are stagnating overall and even declining in some humanities and social science disciplines, which used to be the largest departments at universities, these disciplines are the most affected (Fargahi & Meier, 2024; SAGW, 2023).

Subsequent research should test the robustness and generalizability of our findings. This applies to an extension to other university systems, to topics beyond digitalization, and to other methods of data collection and analysis, including student-level data. The opportunities and risks of new segmentation in HE should also be discussed. Our article provides some initial input for such endeavors.

## Footnotes

1 There are some private universities, but they play a negligible role in the SHES. Therefore, we focus on public universities.

2 Geographical location is particularly important for students from less privileged backgrounds, from peripheral rural areas, and for students at UTE (Denzler & Wolter, 2010).

3 There are some differences in funding according to the university type: For example, CRUs receive around 12% of their global budget from the federal government, around 50% from the cantons, 20% from competitive research funding (mostly SNSF and ERC grants), and 7% from private third-party funding. Tuition fees cover a very small part (2%) of the total costs. At most CRUs, they are currently between CHF 1000 and 1700 per academic year (SKBF 2023).

4 See online: https://www.dizh.uzh.ch/en/about-us/ (access: 20.03.2025).

5 This includes degree programs in computer sciences, business informatics, and communication systems. See https://www.bfs.admin.ch/bfs/de/home/statistiken/kultur-medien-informationsgesellschaft-sport/informationsgesellschaft/gesamtindikatoren/bildungswesen-bibliotheken/ikt-ausbildung.html (accessed: 04.04.2025).

6 Unfortunately, it was not possible to conduct a survey before 2017 because in most universities information about study programs was only partially available for earlier years, which would have significantly impaired the data quality.

7 The following keywords were used: 4.0, AI, artificial intelligen*, computational, computer science, computer-aided, computing, crowd, cyber, data, digital*, information*, intelligen*, machine learning, open, system.

8 A digitalization strategy describes the challenges of digitalization for a university, derives goals from these, systematizes decision-making situations, and formulates routines for achieving the goals (Fernández et al., 2023).

9 We sampled the descriptions of the study programs and used BERTopic models to calculate a uniqueness score. BERTopic is a topic modeling technique that creates dense clusters that render topics easily interpretable while keeping important words in the topic descriptions (Grootendorst 2022). Our uniqueness value ranges from 0 to 1 and measures how dissimilar each program’s description is from the descriptions of the other programs.

## Declarations

### Statement on data access

The dataset (Leder et al., 2024) is available under the following link: https://doi.org/10.48573/JV8D-BF49.
